# Cell quantification in digital contrast microscopy images with convolutional neural networks algorithm

**DOI:** 10.1038/s41598-023-29694-7

**Published:** 2023-02-14

**Authors:** E. K. G. D. Ferreira, D. S. D. Lara, G. F. Silveira

**Affiliations:** 1 Carlos Chagas Institute, Curitiba, PR Brazil; 2 Department of Electrical Engineering, Federal University of Minas Gerais, Belo Horizonte, MG Brazil

**Keywords:** Computational biology and bioinformatics, Cellular microbiology

## Abstract

High Content Screening (HCS) combines high throughput techniques with the ability to generate cellular images of biological systems. The objective of this work is to evaluate the performance of predictive models using CNN to identify the number of cells present in digital contrast microscopy images obtained by HCS. One way to evaluate the algorithm was through the Mean Squared Error metric. The MSE was 4,335.99 in the A549 cell line, 25,295.23 in the Huh7 and 36,897.03 in the 3T3. After obtaining these values, different parameters of the models were changed to verify how they behave. By reducing the number of images, the MSE increased considerably, with the A549 cell line changing to 49,973.52, Huh7 to 79,473.88 and 3T3 to 52,977.05. Correlation analyzes were performed for the different models. In lineage A549, the best model showed a positive correlation with R = 0.953. In Huh7, the best correlation of the model was R = 0.821, it was also a positive correlation. In 3T3, the models showed no correlation, with the best model having R = 0.100. The models performed well in quantifying the number of cells, and the number and quality of the images interfered with this predictive ability.

## Introduction

The High Content Screening (HCS) or High Content Analysis (HCA) equipment was developed with the aim of combining the efficiency of high performance techniques with the ability to collect quantitative data from cellular images of complex biological systems^1^. HCS is a type of automated microscopy capable of acquiring and analyzing fluorescence or light field (digital contrast) images for multiparametric evaluations of cellular assays on microplates. Possible applications of this microscopy include evaluations of cell morphology, cell death, nuclear morphology, internalization of membrane proteins and others^2^. These technological advances have been observed in the last two decades, bringing a constant flow of information, data and insights previously hampered by technical limitations^3^.

Significant technological advances have enabled high throughput microscopy in HCS. Improvements were made in the extraction of quantitative measurements from the acquired images, facilitated by the evolution of the image analysis software^4^. Working with microscopy images also requires the management and interpretation of terabyte scale data of images generated by analysis algorithms, which requires increasingly robust and sophisticated solutions. In addition to integrated software platform solutions, including statistical analysis, computational training methods have recently emerged to automatically score unusual cell morphologies and access information network and databases using commercial and/or opensource components^4^. Along with the equipment comes the Harmony software, which has a simple workflow, allowing the visualization of phenotypes, even in complex cell models^5^.

### Digital phase contrast/brightfield image processing

The Highyield digital contrast experiments are more demanding than fluorescence image processing, presenting several difficulties. Cells captured by digital contrast microscopy have heterogeneous intensity levels and are often poorly contrasted. Furthermore, differences in illumination over time and in the cell culture plate hinder the ability to specify a set of parameters for algorithms during cell detection throughout the experiment. This lighting hampers the application of automatic image processing structures that are already available but are developed mainly for fluorescent images, where they present satisfactory performance^3^. To account for the number of cells analyzed by the Harmony software, one of the strategies may be determining the intensity of a fluorescent marker in the nucleus and the capture of staining using a nuclear marker that is impervious to the membrane for cell viability tests, quantification of DNA content in Fluorescence In Situ Hybridization (FISH) tests, and expression of a nuclear protein or with DAPI marker^6^. Cell quantification is used in many HCS applications, representing an essential reading in Cellular and Molecular Biology research. Determining the count of a cell population is a sensitive indicator of cell stress since cell proliferation requires intact cell structures and functions, and often fluorescence can be toxic to cell culture^3^. Extensive illumination for marker excitation can be harmful to cells due to thermal and/or photochemical effects, such as generation of reactive oxygen species (ROS) via fluorescent label excitation^7,8^. These effects cause stress to cells in different studies, potentially making the information obtained with data and time-lapse images misleading or useless^9^. At very high light intensities, which are necessary, for example, in fluorescence recovery after photobleaching studies, light spread by illuminated cells can induce phototoxic effects in neighboring cells that are not illuminated^10^.

### Machine learning

**Figure 1:**
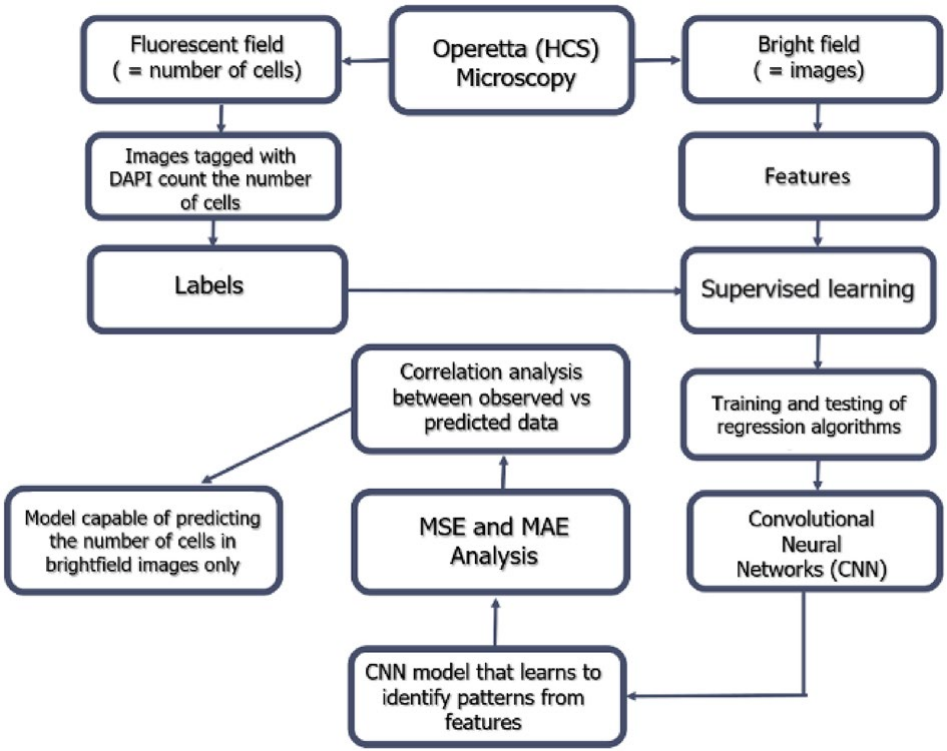
Data processing flowchart.

Machine Learning (ML) is a subarea of AI and Data Sciences, which aims to submit data to a computer that will perform a learning process through computational algorithms^11^. This technique aims to offer a general solution, learning the characteristics of processing rules from examples, other than relying on manual adjustments of parameters or predefined processing steps^12–14^. This method is particularly superior to conventional image processing programs when it comes to solving complex multidimensional data analysis tasks, such as discriminating morphologies that are not easily described by some parameters^15–17^. ML types can be separated into different tasks: supervised (classification and regression), unsupervised, and reinforcement. For the analyses, we aimed to develop and analyze the performance of computational models using different ML algorithms in regression problems to quantify cells in digital contrast images. For this purpose, an action pipeline was followed (see description in Fig. 1). In this case, supervised learning was used because the data were previously labeled with entries to quantify the number of cells provided by the native Operetta (Harmony) software, considered the gold standard of the technique.

### Convolutional neural network

The use of a Convolutional Neural Network (CNN) has performance advantages in some ML problems. The results can be surprising, especially when CNN is applied to image data, computer vision, and natural language processing (NLP)^18^.

A neural network is a structure that behaves similarly to a human brain and the way it learns. The brain receives the stimulus from the outside world and performs the processing, generating a result. As the task becomes more complex, several neurons form a complex network, transmitting information to each other. The information moves from the input layer and to the hidden layers. Hidden layers process and send the final output to the output layer^19^.

In recent decades, CNN has demonstrated positive and innovative results in various fields related to pattern recognition when employed in image recognition. Another important aspect of CNN is the possibility of obtaining different abstract features from the data characteristics when the input propagates toward the deeper layers of a network. The values of each pixel in the image are grouped with neighboring pixels through the application of a permutation invariant (Kernel) function, which helps to reduce the dimensionality of the convolutional layers. The benefit of this architecture is that the CNNs search for spatial dependencies in the image and consider only a local neighborhood for each neuron, that is, the network parameters are shared so that the network performs convolution operations on the images^20^. For example, in image classification, the border can be detected on the first layers and then the simplest shapes on the second layers, and then the top level features^18^.

According to the type of task to be used, the loss function and accuracy assessment can be divided into learning classification (to predict classes) and regression (to predict values). The loss functions to evaluate the classification task that are often used are hinge loss and cross entropy^21^. In this case, as the objective of the work is the cell count, the task used is regression. The most used evaluation is mean absolute error loss (MAE) and mean squared error loss (MSE)^20^.

### Related work

The use of CNN applied to biological problems is a common practice and has recently been used for classifying cell images and quantifying cell nuclei^22^. This method is an automated alternative to classify or quantify cells in microscopy images to guarantee a better result since most techniques are performed in manual counting, a tedious and error prone process^23^. Khan, Gould and Salzmann (2016)^23^ proposed models for counting embryonic cells using CNN. Their results demonstrate that the applied approach surpasses four methods different from other studies, mainly in the quantification of cells at the initial stage of human embryo development.

Kang et al. (2020)^24^ established a CNN model to quantify cells based on images to predict “responses of glioblastoma cells to a drug using automatic image processing,” comparing the model with manual methods. The authors concluded that CNN was more effective compared to manual counting. Loh et al. (2021)^25^ applied a Mask R-CNN deep learning model to cell imaging to identify healthy and Plasmodium-infected red blood cells and compared it to the manual method. They concluded that the proposed model is 15 times faster than the manual method and that, after standardization, it can be an ally in reducing errors resulting from manual counting.

This paper aims to evaluate the accuracy of predictive models from ML algorithms in the task of identifying the number of cells present in digital contrast microscopy images.

## Results

### Evaluation of mean square error (MSE) during training and testing

#### MSE decreases with each round of model training and testing of the A549 cell line images.

**Figure 2:**
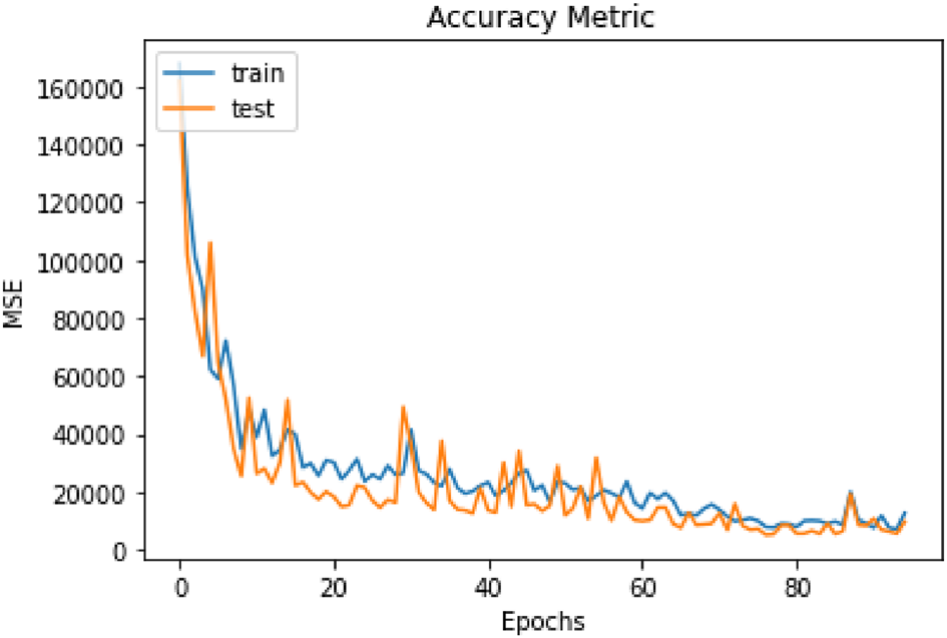
Training rounds compared to the A549 imaging test. In orange, the test line is observed, and in blue, the training. The MSE value decreases (y-axis) after each training round (x-axis).

After 191 rounds of model training, the MSE was calculated by analyzing the number of cells predicted by the model in the training and test bench. As the training rounds progress (x-axis) in cell line A549 (Fig. 2), the MSE value (y-axis) decreased, both in the test database (orange line) and training database (blue line). At the end of the training rounds, the model had an MSE of 4,335.99.

#### MSE decreases with each training round and testing the model in Huh7 cell line images.

**Figure 3:**
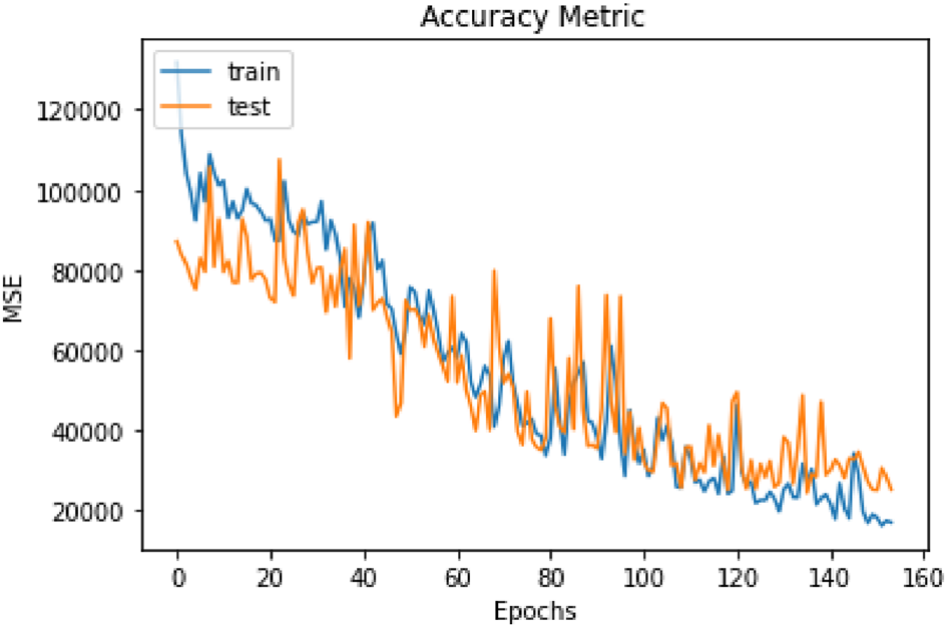
Training rounds compared to testing in Huh7 images. In orange, the test line is observed, and in blue, the training. The MSE value decreases (y-axis) after each training round (x-axis).

For the Huh7 image bank, 154 rounds of model training and the same calculation of the MSE were performed. In Fig. 3, the MSE in the model has a constant decline since the beginning of the training, apparently more homogeneously than occurred in the A549 cell line. With each training round, the MSE (y-axis) decreases. The model had an MSE value of 25,295.23.

#### MSE has different values during the model training and testing in images of the 3T3 cell line.

**Figure 4:**
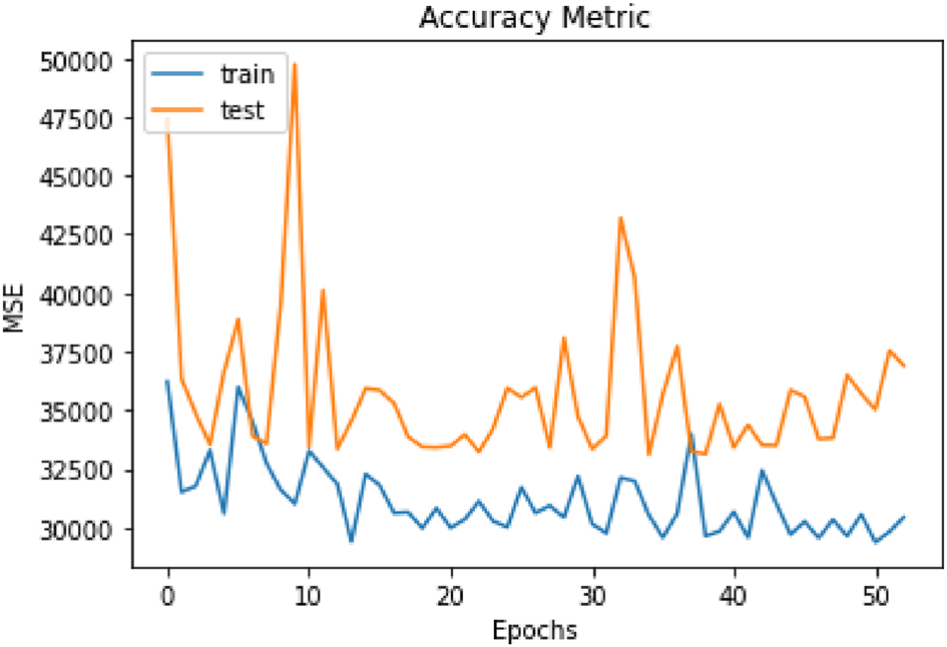
Training rounds compared to the 3T3 imaging test. In orange, the test line is observed, and in blue, the training. From each training round (x-axis), the MSE value dis not synchronized between test and training and remains at constant values (y-axis).

No MSE reduction was observed in the model training with the images of the 3T3 cell line during the 61 rounds, indicating that the model was not able to find a pattern in these images. Additionally, the difference in MSE between the training (blue) and test (orange) banks did not present concomitances, reinforcing that the model failed to understand the images in the different databases (Fig. 4).

### MSE value variations in different parameters

#### MSE value changes as parameters are modified

To evaluate the accuracy of the CNN model in different scenarios, the size of the training/test database was changed to compare the performance of the model. Table 1 shows the parameters used in all tests that were not changed.

**Table 1:** Parameters changed to evaluate the accuracy of the CNN model.

Layers	4
Kernel Size	3
Activation Function	ReLU
Pool Size	2,2
Strides	2,2
Padding	Valid
Dropout	90%

Compilation of each MSE result shows that the MSE value was reduced when the A549 image database was increased to 1137 images, with an average error of 4,335.99. Compared to the other results, such as the database of only 176 images (equal to the smallest image bank belonging to the Huh7 cell line), the error value was 49,973.52 (almost 12 times higher) when separated into 40% of testing and 60% training (Table 2).

**Table 2:** Parameters changed to evaluate the accuracy of the CNN model—A549 cell line.

Test	30%	40%	30%	40%	30%
Training	70%	60%	70%	60%	70%
Images	284	284	176	176	1.137
R2 Score	−103.11%	13.74%	−57.8%	−154.57%	95.35%
MAE	148.86	97.47.06	140.63	172.5	50.21
MSE	32,017.55	13,597.93	28,008.42	49,973.52	4,335.99

Table 3 shows the results of the analyses from the huh7 imaging database, with the lowest bank cell line among the three analyzed. The original database comprises 176 images; when separating this data into 40% for training and 60% for testing, the model had the highest MSE value (79,473.88). When increasing the database to 704 images, the model had the lowest MSE value (25,295.23).

**Table 3:** Parameters changed to evaluate the accuracy of the CNN model—Huh7 cell line.

Test	30%	**40%**	**30%**
Training	70%	**60%**	**70%**
Images	176	**176**	**704**
R2 Score	47.04%	**26.56%**	**67.46%**
MAE	199.03	**180.6**	**115.14**
MSE	53,669.86	**79,473.88**	**25,295.23**

In terms of the 3T3 cell line database, the increase in the image bank also resulted in the lowest MSE value (36,897.03), like the previous lineages. When using the bench with the lowest number of images (176) separated into 40% test and 60% training, the MSE increased to 52,977.05 (Table 4).

**Table 4:** Parameters changed to evaluate the accuracy of the CNN model—3T3 cell line.

Test	30%	40%	30%	40%	30%
Training	70%	60%	70%	60%	70%
Images	208	208	176	176	832
R2 Score	0.35%	7.42%	−0.78%	−22.77%	−11.4%
MAE	179.81	172.02	183.19	176.0	134.68
MSE	47,758.71	45,138.19	43,685.29	52,977.05	36,897.03

### Correlation between observed and predicted values

#### The model with the smallest error in the A549 cell line has a positive correlation

**Figure 5:**
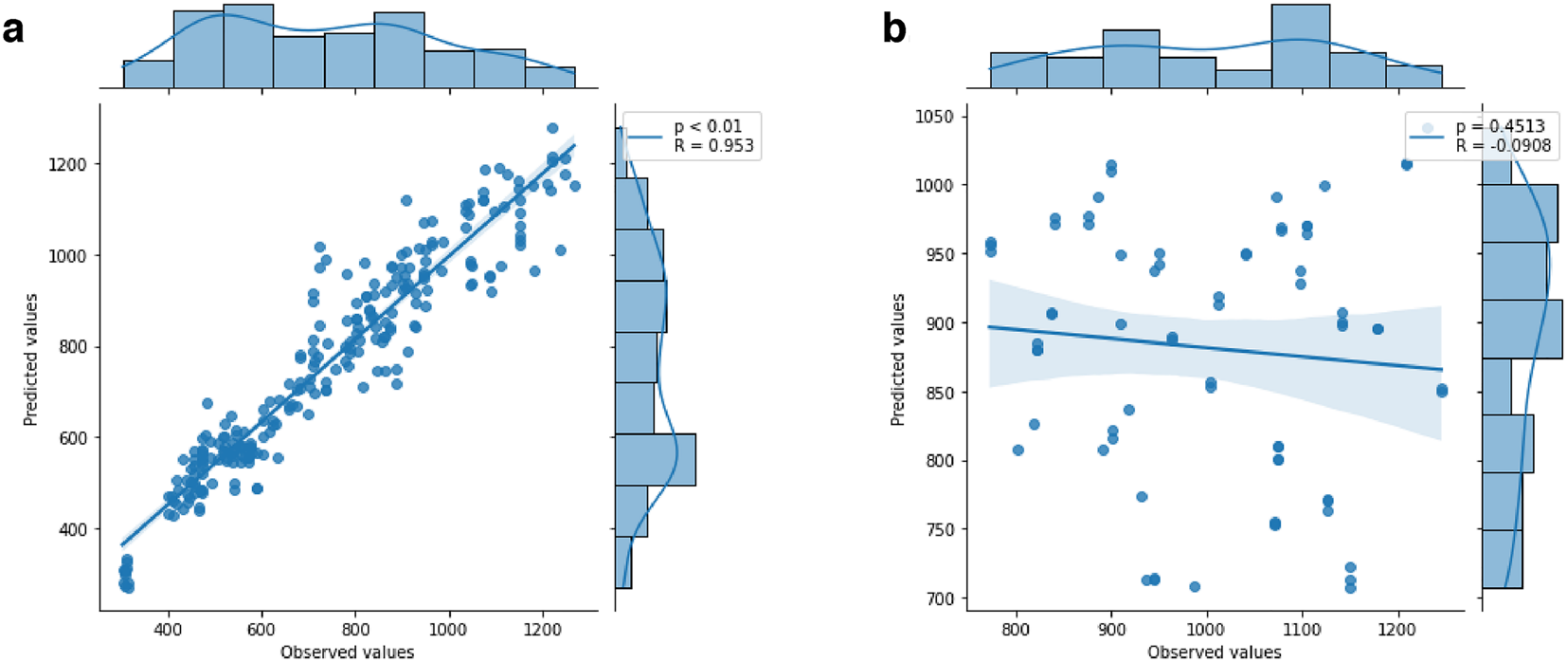
Pearson correlation between predicted and observed values. (**a**) Correlation based on the model that presented lower MSE in the A549 cell line. (**b**) Correlation based on the model that presented higher MSE.

Figure 5 a shows the correlation graph between the observed and predicted values of the number of cells present in the A549 imaging test bench. In this analysis, a value of R = 0.953 can be observed, indicating a significant strong positive correlation (*p* < 0.01 in the Pearson test) between the observed and predicted values. Figure 5b shows the correlation of the model with the highest MSE value, with R = -0.009 and *p* = 0.451 of the Pearson test, showing no correlation.

#### The model with the lowest error in the Huh7 cell line has a moderately positive correlation.

**Figure 6:**
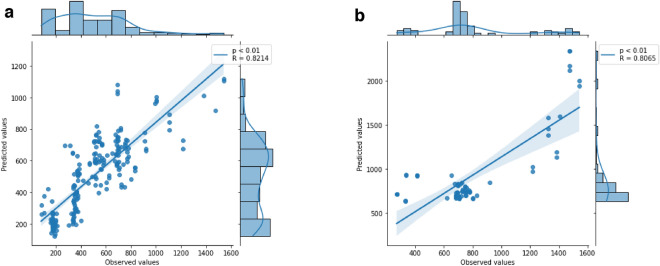
Pearson correlation between predicted and observed values. (**a**) Correlation based on the model with the lowest MSE in the Huh7 cell line. (**b**) Correlation based on the model with the highest MSE.

The correlation between the observed and predicted values was moderately positive in the Huh7 cell line model, presenting the lowest MSE value (R = 0.821; *p* < 0.01 in the Pearson test; Fig. 6a). In the model with the highest MSE, the correlation remained moderately positive but with R = 0.806 and *p* < 0.01 (Fig. 6b). Although the R values were close, the model error started at MSE 500.

#### The model that showed the lowest error in the 3T3 cell line did not present a correlation between the values

**Figure 7:**
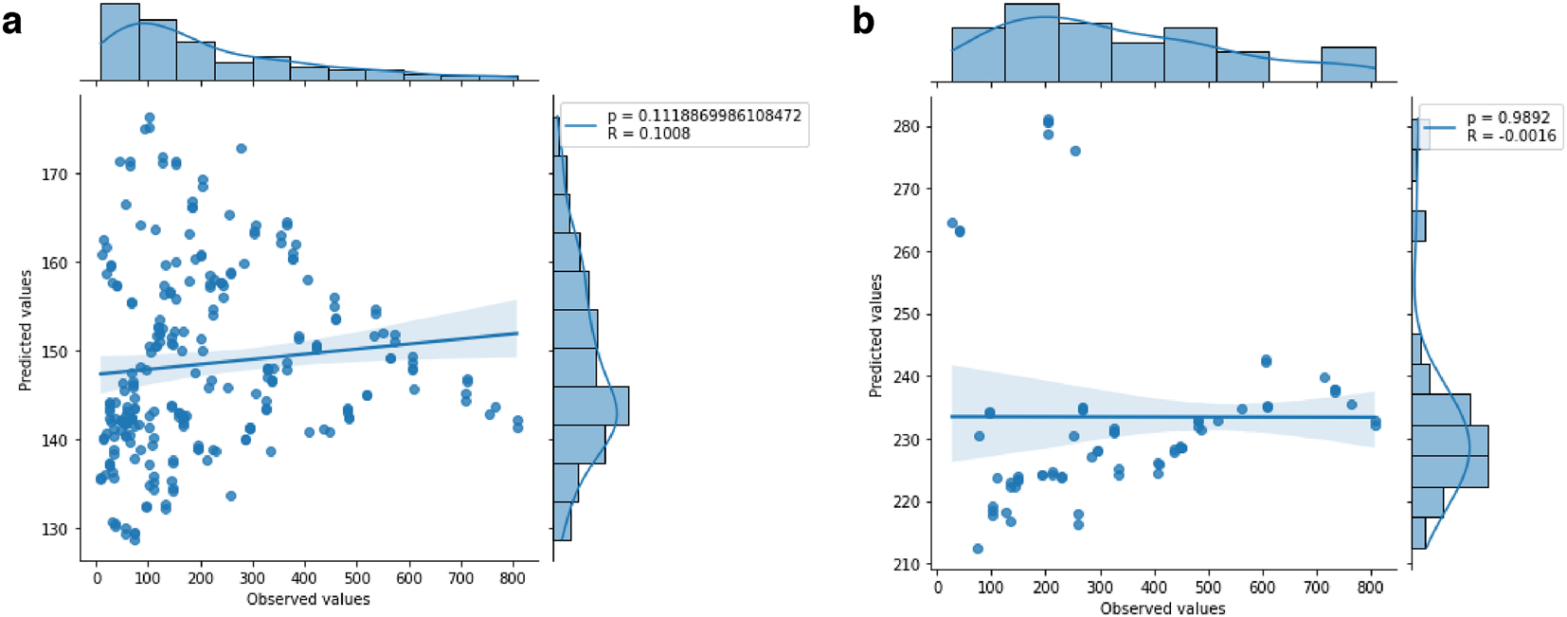
Pearson correlation between predicted and observed values. (**a**) Correlation from the model with the lowest MSE in the 3T3 cell line. (**b**) Correlation based on the model with the highest MSE.

In the model of lower MSE of the 3T3 cell line, it showed a weak positive correlation with R = 0.100 and *p* < 0.01 (Fig. 7a), however, it is observed that the data are asymmetric. In the higher MSE model, it presents no correlation with R = −0.001 and *p* = 0.982 in the Pearson test (Fig. 7b).

## Discussion

In the present work, we developed prediction models from ML algorithms to quantify the number of cells in digital contrast microscopy images of the HCS Operetta. Considering the analyses of the set of images, we obtained different results in the cell count according to each line. We were able to evaluate the accuracy of the models using MSE and assess different parameters in the construction of the network that were passed to the predictive model. We also found a correlation between predicted values and observed values.

It was observed in different works, the use of machine learning for cell quantification, however, most approaches are for specific biological questions, different from what we propose. A generalist approach that works for different biological questions that use different cell types and different morphologies. As for example, in the article by Xie, Noble and Zisserman^26^, in which the authors present a regression algorithm to quantify cells from cell aggregates and clusters, however, it is necessary that the cells are stained with fluorescence. In the article by the authors Umpon and Gader^27^, who use neural networks to determine the number of cells in bone marrow images, despite performing quantification, they use a classification algorithm and focus only on bone marrow cells.

From the first analysis after building the model, the MSE evaluation showed a decrease in the comparison between the data training and testing of the A549 and Huh7 cell lines, and this can be observed from the correlation analysis when the predicted values are next to the labels. This result suggests that the model constantly configured a capacity for gradual learning of the patterns present in the database images at each new training round.

According to Filho^28^ and Rodrigues^29^, it is necessary to measure the quality of the predictive model according to the objective of the task. There are mathematical functions to assist in evaluation of the ability to error and hit the models. When defining a metric, factors such as the proportion of data and the forecast objective must be considered.

The model had difficulty interpreting the set of images for the 3T3 cell line, probably because they are poorly contrasted. The results suggest that the main difficulty in the 3T3 cell line model is not due to the amount of images used in training and testing the network, but due to the quality of the acquired image. Because it is a lineage of embryonic fibroblasts, it has a high cell density, the authors mention that conventional tissue cell culture is produced on 2D surfaces, obtaining little space for cells to adopt natural morphologies or to be able to communicate efficiently with neighboring cells, moreover, cells can behave very differently depending on the growth substrate employed^30^, which may compromise visualization through brightfield. According to the Operetta PerkinElmer manual^31^, the image background intensity is roughly the same intensity as the cell, allowing only texture-based segmentation methods. In highly confluent monolayer cell lines, ultrafine cell regions and clear field images produce a particularly low signal to noise ratio, which makes it difficult to visualize the structures. Furthermore, the 3T3 cell line has a larger image bank than the Huh7, which ended up performing better, even with a reduced database. The morphological differences presented by the cell lines studied in the present work allow us to observe two points in relation to the possible extrapolation of the CNN as a determinant of the cell quantification prediction model. First, in cells such as A549 and Huh7.5 used in the work, despite being morphologically distinct, it was possible to determine robust models (with a strong positive correlation between observed and predicted). In these cells, the main limiting factor was the number of images. Second, the morphological differences were limiting only when the resolution quality of the images was lower, which we observed in the case of 3T3. Thus, we believe that the present work shows that for the generalization of the modeling technique with CNN, both the quality of the images and the number of images in the bank are factors to be observed.

After evaluating the initial metric of the models by MSE for each cell line, the size of the image database was modified, aiming at a better performance of the algorithms. The models with the lowest MSE were the ones that received the highest number of images, thus obtaining better results. According to Somer and Gerlich^32^, an accurate evaluation of the performance of an ML method needs a comprehensive and representative dataset for the specific goal.

According to Gandhi^33^, having a large dataset and information is crucial for good performance. Jain^33^ indicates that a common obstacle to using deep learning to solve problems is the amount of data needed to train a prediction model. The need for big data arises due to the large number of parameters in the model that machines need to learn.

In the present study, the models that received fewer images (176) presented the highest MSE (A549 cell line 49,973.52; Huh7 cell line 79,473.88; 3T3 cell line 52,977.05).

To confirm the ability of the models to predict the number of cells present in microscopy images, Pearson correlation analysis was performed between the observed value and the predicted value. In the model with the lowest MSE in cell line A549, the correlation was strong positive (R = 0.953), different from what was observed in the model with the highest MSE, which had a null correlation (R = −0.090) between the data. This finding supports the hypothesis that the dataset size should be greater than 1,137 for acceptable algorithm performance according to the proposed configuration.

In the model with the lowest MSE of the Huh7 line, the correlation was moderately strong positive (R = 0.821), with some points close to the line, but also with the presence of discrepant values. The model with the highest MSE value maintained a positive correlation, with several values distant from the cell line (R = 0.806).

In the last 3T3 cell line, the model with the lowest MSE value had a weak positive correlation (R = 0.100). However, it is possible to observe an out of normal distribution, especially in the histogram graphs along the correlation. The model with the highest MSE value had a null correlation (R = −0.001), with most points distant from the line.

## Materials and methods

### Image collection

The criterion for selecting the images was to search for studies with different types of strains, widely used in biomedical research at our institution, and which were available in the Harmony software database. Cell manipulation follows the instructions of the American Type Culture Collection (ATCC).

The A549 lineage are epithelial cells of lung tissue. For processing the cell culture, it was necessary to treat the DMSO culture medium at a temperature of 37 °C, atm 95% and CO^2^ density at 5% at a cell concentration of 6 X 10^3^ and 6 X 10^4^ cell/cm^2^^34^. Huh7 strain are hepatocytes (liver cells), DMEM culture medium needs to be at temperature at 37 °C, CO^2^ density at 5%^35^. The 3T3 cell line is murine fibroblast cells, the culture medium (Eagle) at 37 °C temperature, atm 95% and CO^2^ density at 5% showing cell concentration of e 3 to 5 X 103 cells/cm^2^^36^.

The images used for the database construction were selected from among the projects already analyzed by the Harmony software version 3.5 that has been accompanying the HCS fluorescence microscopy of the Operetta equipment. We searched for experiments that generated digital contrast images. After the responsible researchers signed the consent form, images were selected among the projects. The microscopy images selected for the study are of cell lines A549, Huh7, and 3T3. Brightness and color were also adjusted in Harmony to solve differences in image lighting, seeking to optimize the visibility of cellular nuclei; for this, light contrast adjustments (enhancing nuclear marking) and background correction (setting the background of the image) were performed. As an example, the contrast adjustment of the 3T3 cell line is presented: minimum 4776 /maximum 55,818 (Custom brightness and contrast adjustment); Gamma: 1.0 (the closer to 1.0, the darker the background of the image).

### Data augmentation

A database with a small amount of data for training a neural network can cause low model accuracy. However, to avoid this problem, we used some data augmentation techniques, in which it was possible to expand our database from 167 images to 2,673 images and ensure good performance of the model, where the images were changed in different orientations (vertical and horizontal rotation technique), from left to right from top to bottom, and from bottom to top. In addition to working with images with 100% of their size, a clipping (scaling technique) was performed, reducing 75%, 50%, and 25% of the size of the original images (Fig. 2). These images were resized to 200 × 200 pixels to allow analysis by the algorithm. During collection, the number of cells corresponding to each image from the Operetta was also recorded, which was used as an observed value. This value was then reduced in the same proportion of the images to perform the supervised training of the models and, later, the tests against the predicted values.

#### A549 cell line

**Figure 8:**
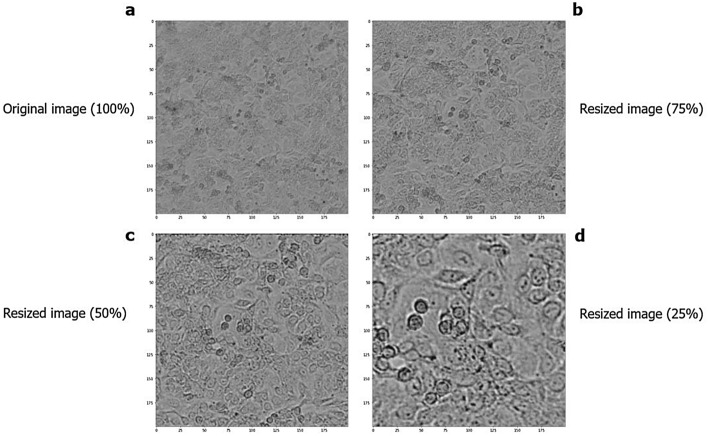
Microscopy images of the A549 cell line in different dimensions. (**a**) Original image. (**b**) Cropping 75% of the image. (**c**) Cropping 50% of the image. (**d**) Cropping 25% of the image.

Seventy-one digital contrast images were selected, with a resolution of 1080 × 1080 pixels. This processing was performed as a strategy to increase the number of images, as described above. The database was enlarged to 1,136 images from the original ones. Figure 8 shows examples of digital contrast images of A549 cultures after scaling.

#### Huh7 cell line

**Figure 9:**
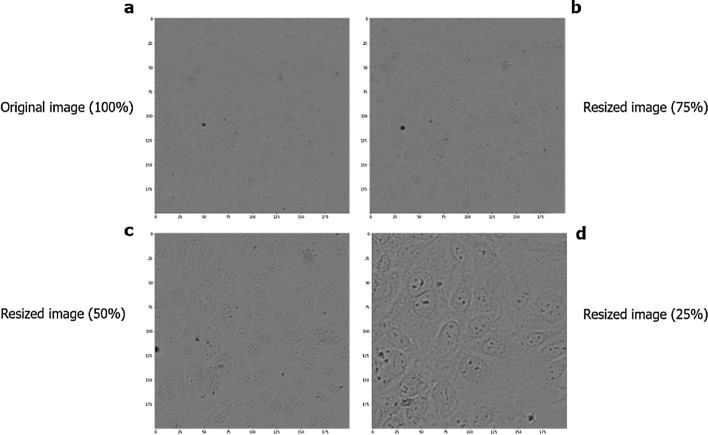
Microscopy images of the Huh7 cell line in different dimensions. (**a**) Original image. (**b**) Cropping 75% of the image. (**c**) Cropping 50% of the image. (**d**) Cropping 25% of the image.

A total of 44 digital contrast images were collected from the Huh7 cell line. After processing, the database increased to 704 images, with the bank with the lowest number of images (Fig. 9).

#### 3T3 cell line

**Figure 10:**
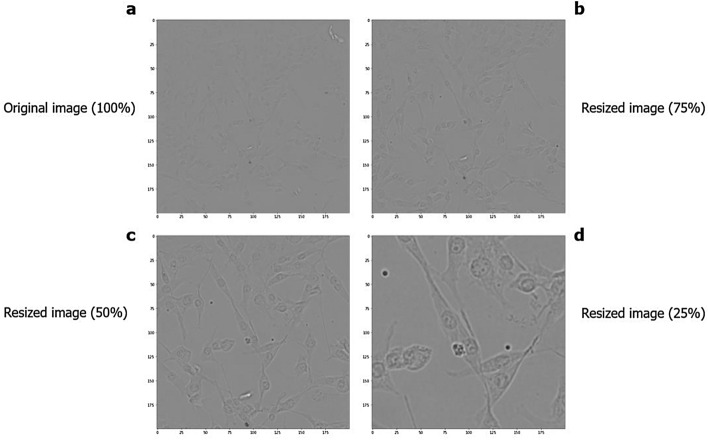
Microscopy images of the 3T3 cell line in different dimensions. (**a**) Original image. (**b**) Cropping 75% of the image. (**c**) Cropping 50% of the image. (**d**) Cropping 25% of the image.

The dataset of the 3T3 cell line comprised 52 original images; after data processing, it increased to 832 images (Fig. 10).

### Algorithm development

Among the algorithms selected for this work, which are similar to the problem, the Deep Neural Networks with CNN architecture was used, which is widely applied in a set of images^37^. The images were randomly separated to form the training database (~ 70%) and test database (~ 30%) of the algorithm. Among the images of the A549 cell line, 795 images were separated for training and 341 images for testing. For huh7 semdage, 492 images were selected for training and 212 for testing. For the 3T3 cell line, 582 images were separated for training and 250 for testing.

### Model determination

The first of CNN's layers (Conv2D), which formats array objects. This layer was initially set to kernel_size = 3 and rectified linear unit (ReLU) activation function. The resulting values were sent as input to the neurons of the next layer, MaxPooling2D. With this new dimension, the set of neurons in the 2D configuration was linearized in the Flatten layer, and from these, a part of the neurons in the Drop out layer was removed, avoiding overfitting the model. This network configuration was repeated with layers of 32, 64, 128 and 256 neurons. As it is a regression model, the last layer of the network was terminated with only one output neuron, using the ReLU activation function representing the number of cells in the image.

### Performance assessment

**Figure 11:**
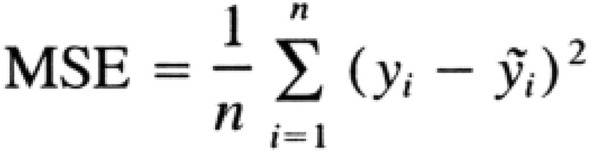
MSE is the $\frac{1}{n}\sum\limits_{i = 1}^{n} {}$ the square of $\left( {Y_{i} - \hat{Y}_{i} } \right)^{2}$.

Different mathematical functions help us to evaluate the ability and degrees of the correct answers and errors of the models from metrics such as Mean Absolute Error (MAE), Mean Square Error (MSE), and Square R, among others^24^. During the training of the model, the Early Stopping method was used, which is responsible for interrupting the training as the model presents difficulty in correcting the number of cells present in the images. In the A549 cell line, the model stopped its training in the 191st round, in the Huh7 cell line the model stopped in the 154th round and in the 3T3, the model ran up to the 61st round. The average training time was approximately 10 min, depending on the infrastructure settings (i.e. memory size and number of processing cores) of the machine used. Soon after, it was possible to analyze the quality of the training of the models, plotting graphs to visualize the performance, with its performance evaluated by the MSE (which calculates the average of the squares of the model errors) (Fig. 11).

### Correlation between observed and predicted values

The values were analyzed using Pearson's test. The null hypothesis (H0) assumed no correlation between the values of the labels in the training and test bench. In contrast, the alternative hypothesis (H1) assumed there is sufficient probability to deny the H0 hypothesis.

## Data Availability

The data and programming code script is available on the GitHub repository, accessible through this link: https://github.com/Laboratorio-de-Analise-de-Dados/doc_CNN_Eloiza.
